# Multidrug resistance and lipopolysaccharide remodeling associated with *mcr-1* acquisition in Shiga toxin-producing *Escherichia coli*

**DOI:** 10.1128/spectrum.02034-26

**Published:** 2026-09-30

**Authors:** Eunbyeol Ahn, Jinshil Kim, Sangryeol Ryu, Byeonghwa Jeon

**Affiliations:** 1 Environmental Health Sciences, School of Public Health, University of Minnesota 5635, St. Paul, Minnesota, USA; 2 Department of Food and Animal Biotechnology, Research Institute of Agriculture and Life Sciences, Seoul National University 26725, Seoul, Republic of Korea; 3 Department of Agricultural Biotechnology, Seoul National University 26725 , Seoul, Republic of Korea; 4 Department of Food and Nutrition, Hanyang University 26716, Seoul, Republic of Korea; Universita degli Studi di Bari Aldo Moro, Valenzano, Bari, Italy

**Keywords:** *mcr-1*, Shiga toxin-producing *E. coli*, lipopolysaccharide, virulence, antibiotic resistance

## Abstract

The emergence of plasmid-mediated colistin resistance in Shiga toxin-producing *Escherichia coli* (STEC) is an important public health concern because these strains can combine antimicrobial resistance with intestinal pathogenic potential. However, although *mcr*-harboring STEC has been reported from food-related sources, the phenotypic consequences of *mcr-1* acquisition in STEC remain poorly understood. In this study, we investigated the prevalence of *mcr* variants and associated antimicrobial resistance determinants among 144 *mcr*-harboring STEC isolates and examined the phenotypic impact of *mcr-1* in *E. coli* O157:H7 ATCC 43894. Among the *mcr*-harboring STEC isolates, *mcr-1* was the predominant *mcr* variant (63.9%), and 88.9% of *mcr-1*-positive isolates were multidrug-resistant. Notably, genomically predicted fosfomycin resistance determinants were detected in 84.8% of *mcr-1*-positive isolates. Acquisition of the *mcr-1*-harboring plasmid in *E. coli* O157:H7 ATCC 43894 increased adhesion to HEp-2 epithelial cells but reduced swimming motility, biofilm formation, and *Galleria mellonella* mortality. The reduced *G. mellonella* mortality may reflect altered innate immune stimulation. These changes were accompanied by remodeling of the lipopolysaccharide profile, with reduced banding in both the O-antigen and core-lipid A regions, reduced endotoxin levels, increased cell surface hydrophobicity, a less negative zeta potential, and reduced autoagglutination, all of which were *mcr-1*-dependent in knockout and complementation experiments. Collectively, these findings indicate that *mcr-1* contributes beyond colistin resistance, converging on a coordinated reshaping of cell surface architecture and host interaction together with co-selection of resistance to other clinically relevant antibiotics, including fosfomycin.

**IMPORTANCE:**

Shiga toxin-producing *Escherichia coli* is a major foodborne pathogen, but treatment options are limited because some antibiotics increase the risk of severe disease. Mobile colistin resistance genes in this pathogen are therefore concerning, especially alongside resistance to other clinically relevant antibiotics. This study shows that *mcr-1* is the predominant colistin resistance gene among *mcr*-harboring Shiga toxin-producing *E. coli* and that these isolates frequently carry multidrug resistance determinants, including fosfomycin resistance. We further show that *mcr-1* acquisition reshapes the bacterial cell surface and alters host-associated phenotypes in *E. coli* O157. Despite reduced mortality in *Galleria mellonella*, increased epithelial cell adhesion suggests that the reduced mortality may reflect altered innate immune stimulation rather than an overall attenuation of host-colonization potential. These findings suggest that *mcr-1* is not only a colistin resistance determinant but also a factor associated with broader changes in resistance, surface architecture, and host interaction in foodborne *E. coli*.

## INTRODUCTION

Colistin is regarded as a last-resort agent for the treatment of infections caused by multidrug-resistant (MDR) gram-negative bacteria (1). However, the emergence and dissemination of mobile colistin resistance (*mcr*) genes have become major public health concerns (2, 3). MCR reduces colistin susceptibility by modifying lipid A with phosphoethanolamine, thereby decreasing the binding affinity of colistin to the bacterial outer membrane (3, 4). To date, *mcr-1* to *mcr-10* have been described, and these genes are widely distributed among gram-negative bacteria, particularly Enterobacterales such as *Escherichia coli*, *Salmonella enterica*, and *Klebsiella pneumoniae* (2, 5). Among them, *mcr-1* is the most widely reported variant, and *E. coli* is one of the major bacterial hosts most frequently associated with *mcr* carriage (2, 6). Since its first description in 2015 (4), *mcr-1* has been detected in isolates from humans, animals, foods, and environmental sources across multiple countries, underscoring its relevance to both clinical medicine and food safety (6, 7).

Shiga toxin-producing *Escherichia coli* (STEC) is an important zoonotic foodborne pathogen that can cause watery diarrhea, hemorrhagic colitis, and life-threatening hemolytic uremic syndrome (HUS) (8). Among STEC, *E. coli* O157:H7 remains one of the most clinically important serotypes because it is frequently associated with outbreaks and severe human disease (8, 9). Ruminants are recognized as the major reservoirs of STEC, and human infection is primarily linked to direct contact with infected animals or the consumption of contaminated food and water (8, 10). Major food vehicles associated with STEC outbreaks include raw or undercooked ground meat, raw milk, and fecally contaminated vegetables and sprouts, highlighting the importance of STEC as a major food safety hazard (8, 9).

The emergence of antimicrobial resistance (AMR) in STEC is particularly concerning because treatment options for STEC infection remain limited, and the use of antibiotics is controversial owing to the potential induction of Shiga toxin (Stx) production (11, 12). In this restricted therapeutic landscape, fosfomycin has been considered a comparatively favorable option because it acts through an SOS-independent mechanism and has been associated with reduced progression to HUS when used early in clinical reports (13). Recent studies have identified *mcr-1*-positive STEC in food-producing animals, beef, and other sources across the food supply chains (14, 15). *mcr-1*-positive STEC have also been reported from raw meat (16), indicating that colistin resistance and STEC-associated virulence may coexist in strains relevant to foodborne transmission (14, 16, 17). Nevertheless, these studies have largely focused on the occurrence, antimicrobial resistance profiles, and genomic characteristics of such isolates, whereas direct analyses of the phenotypic consequences of *mcr-1* acquisition in an STEC background remain limited (14, 16). We have previously shown that an *mcr-1*-harboring plasmid promotes a transition from a rough to a smooth lipopolysaccharide (LPS) phenotype in enterotoxigenic *E. coli* through plasmid-mediated upregulation of surface polysaccharide biosynthesis (18). Whether and how *mcr-1* acquisition affects the surface architecture of STEC, which already produces a smooth, O-antigen-bearing LPS, has not been examined.

In this study, we investigated the distribution of *mcr* variants and associated antimicrobial resistance genes among *mcr*-harboring STEC isolates retrieved from public genomic databases (*n* = 144). We further examined the phenotypic impact of *mcr-1* using *E. coli* O157:H7 ATCC 43894 as a model STEC strain. Specifically, we evaluated whether acquisition of an *mcr-1*-harboring plasmid alters bacterial adhesion, motility, biofilm formation, virulence, and cell surface properties, including the lipopolysaccharide profile, endotoxin level, hydrophobicity, surface charge, and autoagglutination.

## RESULTS

### Distribution of *mcr* genes among STEC isolates

**Fig 1:**
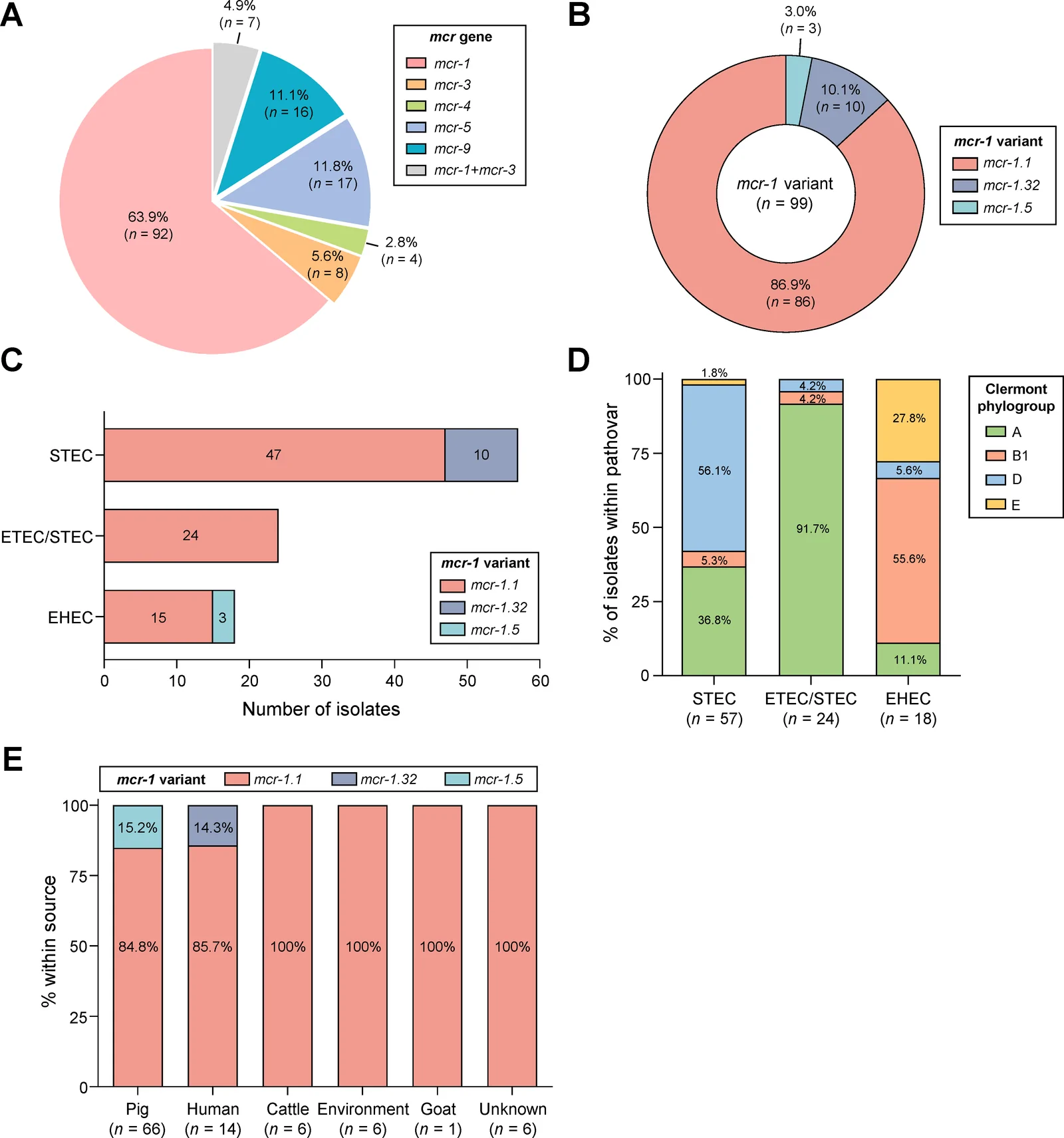
Distribution of *mcr* genes and *mcr-1* variants among *mcr*-harboring STEC isolates. (**A**) Overall distribution of *mcr* gene types among all *mcr*-harboring STEC isolates (*n* = 144). Percentages and isolate numbers are shown for each category. (**B**) Distribution of *mcr-1* variants among *mcr-1*-positive isolates (*n* = 99). The total number of isolates is shown in the center of the donut chart, and percentages and isolate numbers are indicated for each variant. (**C**) Numbers of isolates carrying each *mcr-1* variant according to pathotype category (STEC, ETEC/STEC, and EHEC). Numbers in the bars indicate isolate counts. (**D**) Clermont phylogroup (A, B1, D, and E) composition of *mcr-1* variant isolates within each pathotype category. Percentages indicate the proportion of isolates within each pathotype, and sample sizes are shown below each category. (**E**) Distribution of *mcr-1* variants by isolation source. Percentages indicate the proportion of isolates within each source category, and sample sizes are shown below each category.

To investigate the prevalence and distribution of *mcr* genes among STEC isolates, we analyzed 144 *mcr*-harboring STEC isolate genomes retrieved from the NCBI Pathogen Detection database (Table S2). These isolates harbored diverse *mcr* families, including *mcr-1*, *mcr-3*, *mcr-4*, *mcr-5*, and *mcr-9*, as well as co-harboring *mcr-1* and *mcr-3* (Fig. 1A). Among them, isolates harboring *mcr-1* were the most prevalent (*n* = 92, 63.9%), followed by *mcr-5* (*n* = 17, 11.8%) and *mcr-9* (*n* = 16, 11.1%) (Fig. 1A). Given the predominance of *mcr-1*, we further analyzed isolates harboring *mcr-1*, including isolates carrying *mcr-1* and those co-harboring *mcr-1* with other *mcr* genes (*n* = 99) (Fig. 1B). Three *mcr-1* variants, *mcr-1.1*, *mcr-1.32*, and *mcr-1.5*, were identified, with *mcr-1.1* being the dominant variant (*n* = 86, 86.9%), followed by *mcr-1.32* (*n* = 10, 10.1%) and *mcr-1.5* (*n* = 3, 3.0%) (Fig. 1B).

The *mcr-1*-harboring isolates were further classified into STEC, ETEC/STEC, and EHEC groups based on the presence of *stx* genes (*stx1* and/or *stx2*), enterotoxin genes (*st* and/or *lt*), and *eae* (Fig. 1C and Table S2). In the STEC group, *mcr-1.1* (*n* = 47) and *mcr-1.32* (*n* = 10) were detected, whereas all ETEC/STEC isolates carried *mcr-1.1* (*n* = 24) (Fig. 1C). In the EHEC group, *mcr-1.1* (*n* = 15) and *mcr-1.5* (*n* = 3) were identified (Fig. 1C). Analysis of Clermont phylogroups showed distinct phylogenetic distributions among the three groups. The STEC group was mainly assigned to phylogroups D (56.1%) and A (36.8%), whereas ETEC/STEC isolates were predominantly assigned to phylogroup A (91.7%). In contrast, EHEC isolates were mostly distributed in phylogroups B1 (55.6%) and E (27.8%) (Fig. 1D).

We next examined the source distribution of *mcr-1* variants. The *mcr-1*-harboring isolates were mainly derived from pigs (*n* = 66), followed by humans (*n* = 14), cattle (*n* = 6), environmental sources (*n* = 6), goats (*n* = 1), and unknown sources (*n* = 6) (Fig. 1E). In cattle, environmental, goat, and unknown-source categories, *mcr-1.1* was the only detected *mcr-1* variant (Fig. 1E). In pigs, *mcr-1.1* accounted for 84.8% of isolates, while *mcr-1.5* accounted for 15.2%. In humans, *mcr-1.1* accounted for 85.7%, and *mcr-1.32* accounted for 14.3% (Fig. 1E).

### Antimicrobial resistance profiles of *mcr-1* variant STEC isolates

**Fig 2:**
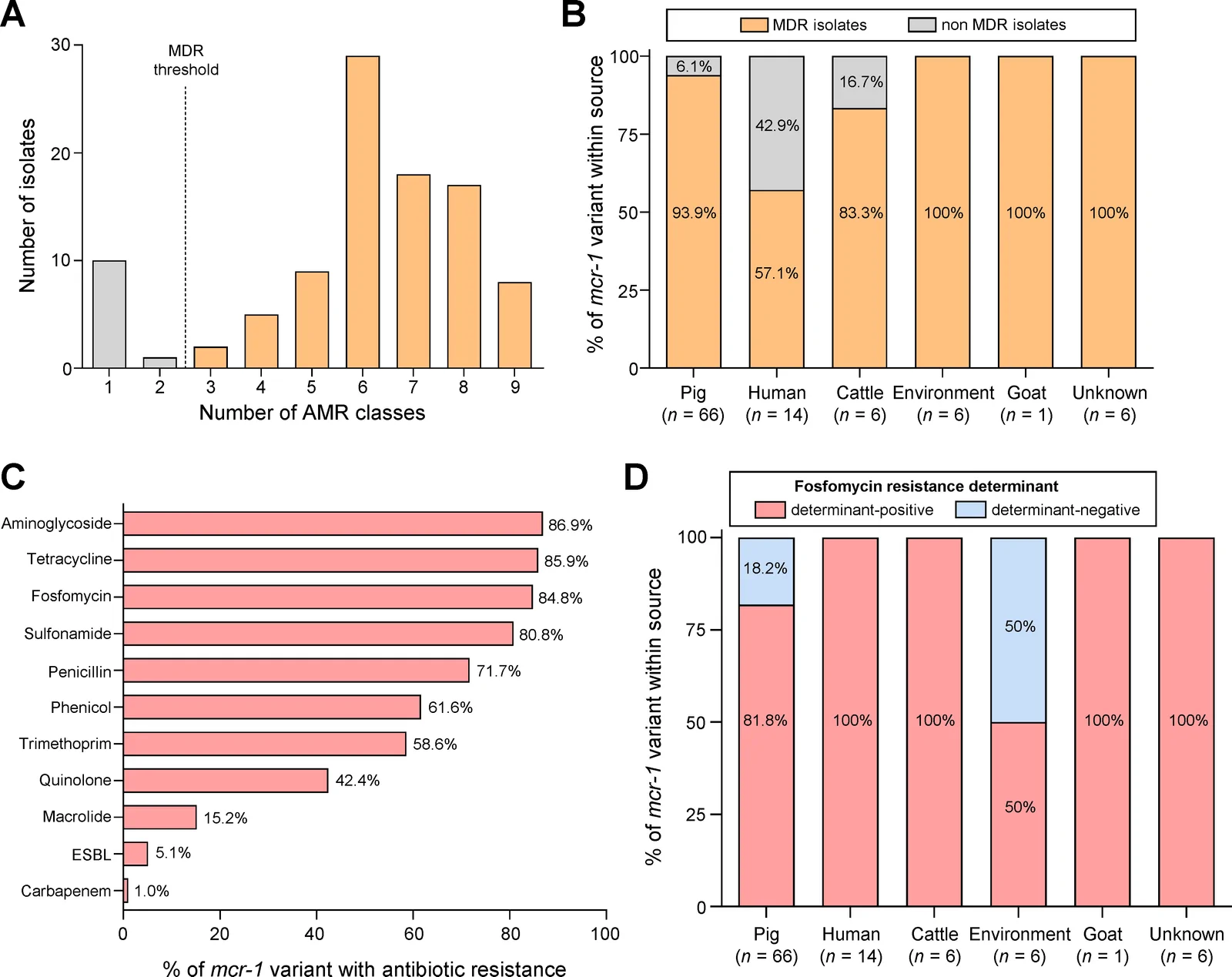
Antimicrobial resistance profiles of *mcr-1* variant isolates. (**A**) Distribution of *mcr-1* variant isolates according to the number of AMR classes detected per isolate. The dashed vertical line indicates the MDR threshold, defined as resistance to three or more AMR classes. (**B**) Distribution of MDR and non-MDR isolates among *mcr-1* variant isolates according to isolation source. Percentages indicate the proportion of isolates within each source category, and sample sizes are shown below each category. (**C**) Frequency of resistance determinants associated with each antimicrobial class among *mcr-1* variant isolates. Percentages are shown at the end of each bar. (**D**) Distribution of any fosfomycin resistance determinant (chromosomal *glpT/uhpT* substitutions or plasmid-borne *fos* genes) among *mcr-1* variant isolates based on isolation source. Percentages indicate the proportion of isolates within each source category, and sample sizes are shown below each category.

We analyzed AMR gene profiles among the *mcr-1* variant isolates (Table S2). A total of 11 AMR categories were identified, including aminoglycosides, tetracyclines, fosfomycin, sulfonamides, penicillins, phenicols, trimethoprim, quinolones, macrolides, ESBL, and carbapenemases (Table S2). Based on the criterion of resistance to three or more antimicrobial classes, 88.9% (88/99) of the isolates were classified as MDR (Fig. 2A). Source-specific analysis showed that the proportion of MDR isolates was 93.9% in pig isolates, 57.1% in human isolates, 83.3% in cattle isolates, and 100% in environmental, goat, and unknown-source isolates (Fig. 2B).

Resistance determinants associated with aminoglycosides (86.9%), tetracycline (85.9%), fosfomycin (84.8%), and sulfonamides (80.8%) were frequently detected (Fig. 2C). Genes associated with penicillin, phenicol, trimethoprim, quinolone, and macrolide resistance were also identified at variable frequencies (Fig. 2C and Table S2). In contrast, ESBL-associated genes (5.1%) and carbapenemase-associated genes (1.0%) were detected only in a small proportion of isolates (Fig. 2C). To further characterize fosfomycin resistance among the *mcr-1* variant isolates, we examined the distribution of fosfomycin resistance determinants in detail (Table S2). The most prevalent determinants were chromosomal substitutions in GlpT and UhpT. GlpT_E448K was detected alone in 48 isolates (48.5%) and in combination with UhpT_E350Q in 30 isolates (30.3%), while UhpT_E350Q alone was detected in 3 isolates (3.0%) (Table S2). Primarily plasmid-mediated fosfomycin-modifying enzyme genes were detected at low frequency, with *fosA3* in one isolate, *fosA7.5* in one isolate, and *fosA* co-occurring with GlpT_E448K in one isolate (3.0% combined) (Table S2). When the distribution of any fosfomycin resistance determinant was analyzed by source, determinants were detected in 81.8% of pig isolates, 100% of human, cattle, and goat isolates, and 50.0% of environmental isolates (Fig. 2D). Collectively, these findings show that *mcr-1* variant isolates frequently carried AMR determinants spanning multiple antimicrobial classes, with a high proportion meeting the definition of MDR. Given the predominance of *mcr-1.1* among the identified *mcr-1* variants, subsequent experiments focused on *mcr-1* to examine whether acquisition of an *mcr-1*-harboring plasmid, and *mcr-1* itself, modulates bacterial phenotypes related to virulence-associated traits, cell surface properties, and colistin resistance.

### Acquisition of the *mcr-1* plasmid alters bacterial adhesion, motility, biofilm formation, and virulence

**Fig 3:**
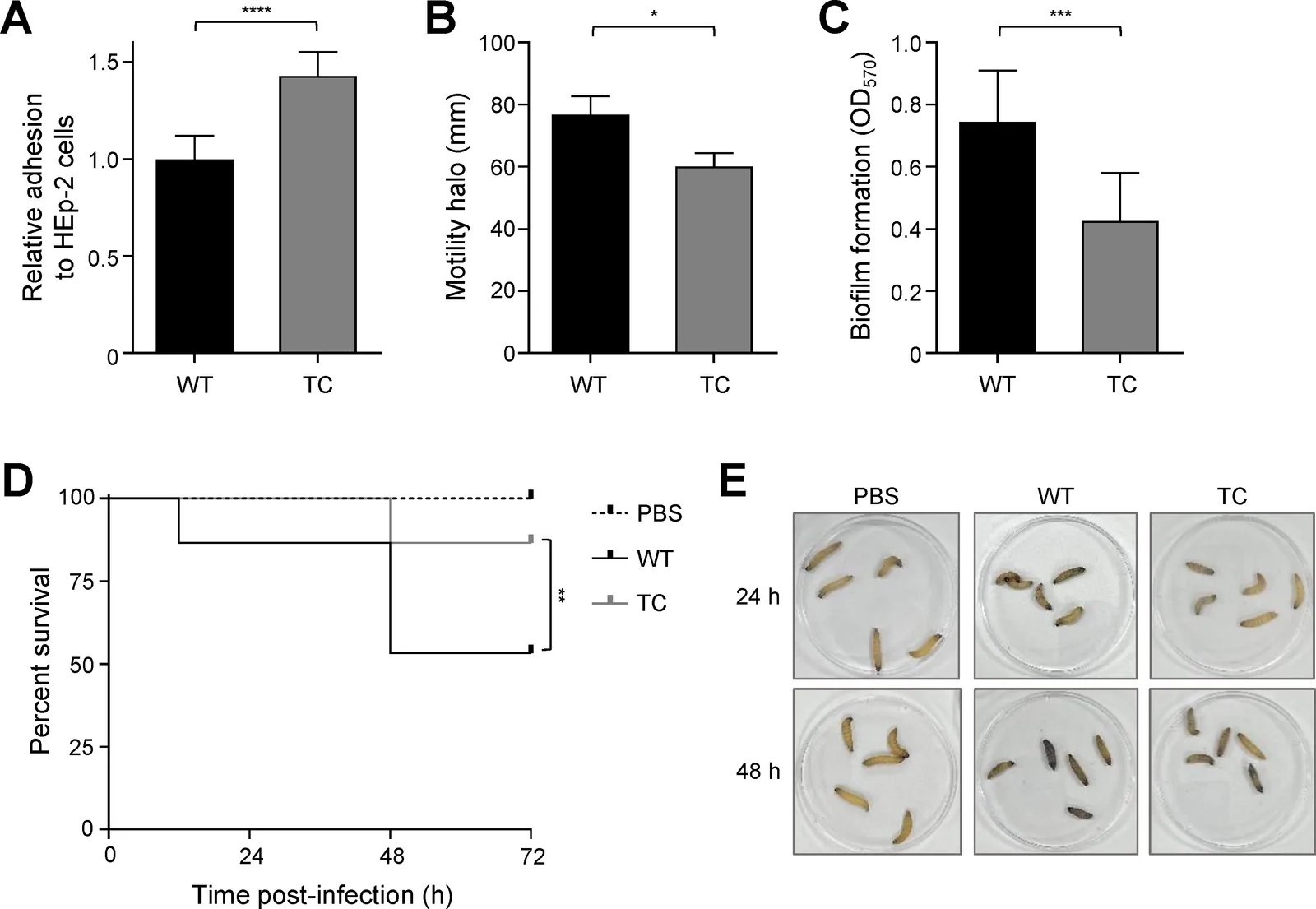
Acquisition of the *mcr-1*-harboring plasmid alters adhesion, motility, biofilm formation, and virulence in *E. coli* O157:H7 ATCC 43894. (**A**) Adhesion of the wild-type strain (WT) and transconjugant (TC) strains to HEp-2 cells. HEp-2 monolayers were infected with bacteria at a multiplicity of infection (MOI) of 10 and incubated for 3 h at 37°C. Adhesion is presented relative to that of the WT. (**B**) Swimming motility of WT and TC. Motility was quantified by measuring the diameter of the motility halo after incubation at 37°C for 30 h. (**C**) Biofilm formation of WT and TC. Biofilm biomass was quantified by crystal violet staining after incubation at 37°C for 72 h. (**D**) Survival of *Galleria mellonella* larvae after infection with WT or TC. Larvae were injected with 10 μL of bacterial suspension (1 × 10^6^ CFU/mL) into a proleg and incubated at 37°C. Survival was monitored for 72 h. Larvae injected with PBS served as the negative control. Survival curves were analyzed using the Kaplan–Meier method and compared using the log-rank (Mantel–Cox) test. **, *P* < 0.01. (**E**) Representative images of melanization in *G. mellonella* larvae at 24 and 48 h after injection with PBS, WT, or TC. Data represent the mean ± standard deviation from three independent experiments. Statistical significance in panels **A–C** was determined using an unpaired two-tailed *t*-test. *, *P* < 0.05; ***, *P* < 0.001; ****, *P* < 0.0001.

Because bacterial adhesion, biofilm formation, and swimming motility are important traits related to host interaction and bacterial fitness in *E. coli*, we compared these phenotypes between the wild-type strain (WT) and the transconjugant (TC). The transconjugant showed significantly increased adhesion to HEp-2 epithelial cells compared with the wild-type strain, indicating that acquisition of the *mcr-1* plasmid enhanced host-cell attachment (Fig. 3A). In contrast to the increased adhesion phenotype, the transconjugant exhibited significantly reduced swimming motility compared with the wild-type strain (Fig. 3B). Biofilm formation was also significantly decreased in the transconjugant, as shown by the lower crystal violet staining intensity compared with the wild-type strain (Fig. 3C).

We further evaluated the effect of *mcr-1* plasmid acquisition on *in vivo* virulence using a *G. mellonella* infection model. Larvae infected with the wild-type strain showed greater mortality over time, whereas larvae infected with the transconjugant showed higher survival rates throughout the infection period (Fig. 3D). Consistent with the survival data, larvae infected with the wild-type strain displayed more pronounced melanization at 24 and 48 h post-infection, while larvae infected with the transconjugant showed less severe melanization (Fig. 3E). Together, these results indicate that acquisition of the *mcr-1* plasmid differentially affected bacterial phenotypes, increasing epithelial cell adhesion while reducing motility, biofilm formation, and virulence in the *G. mellonella* model.

### *mcr-1* mediates reduced swimming motility and biofilm formation

**Fig 4:**
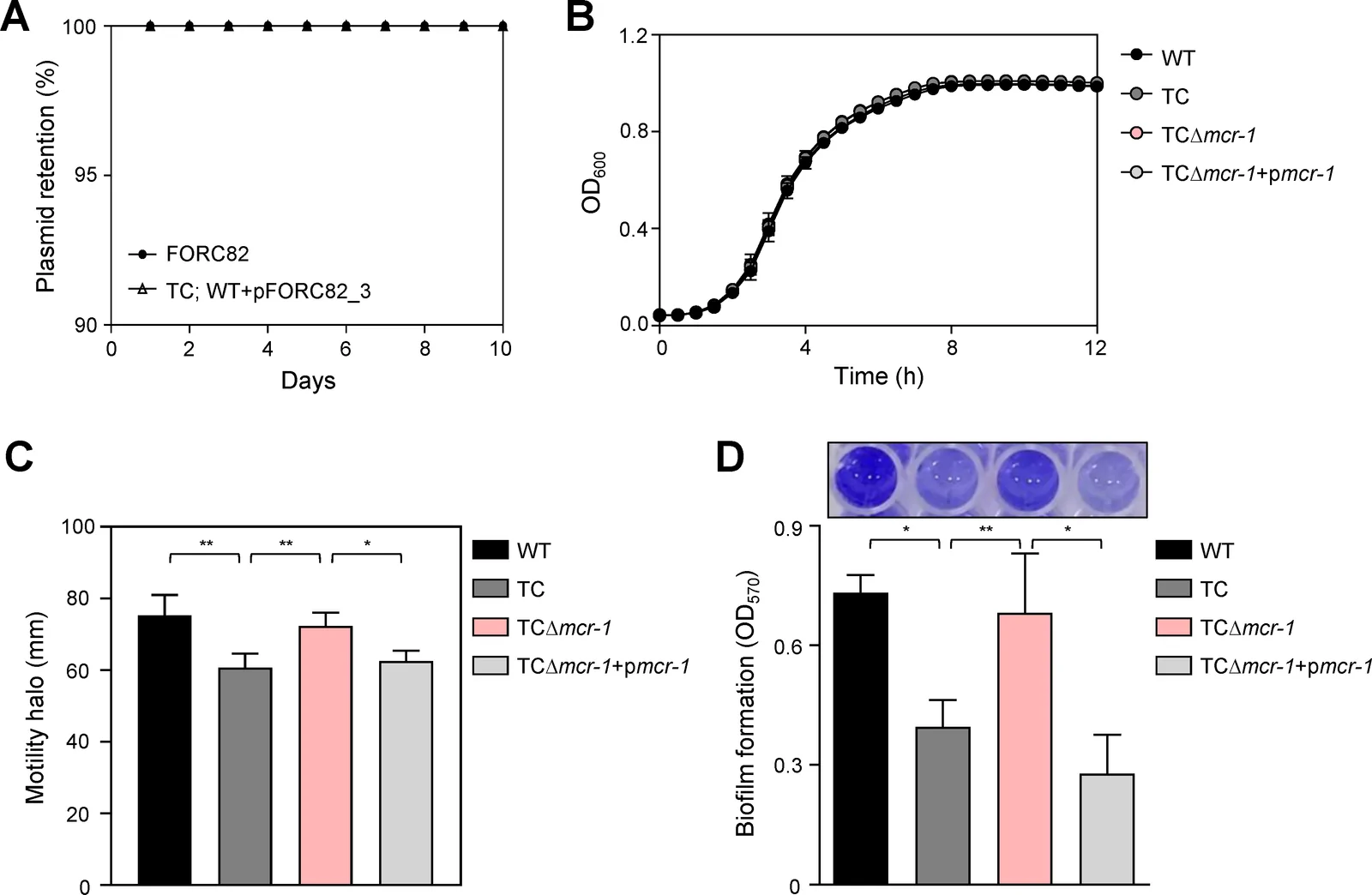
Stability of the *mcr-1* plasmid and contribution of *mcr-1* to motility and biofilm formation in *E. coli* O157:H7 ATCC 43894. (**A**) Stability of the *mcr-1* plasmid in *E. coli* O157:H7 ATCC 43894. FORC82, which originally harbors the *mcr-1* plasmid, was used as the control strain. (**B**) Growth curves of the wild-type strain (WT), transconjugant (TC), Δ*mcr-1* mutant (TCΔ*mcr-1*), and *mcr-1* complemented strain (TCΔ*mcr-1*+p*mcr-1*). Bacterial growth was monitored by measuring OD_600_ for 12 h. (**C**) Swimming motility of WT, TC, TCΔ*mcr-1*, and TCΔ*mcr-1*+p*mcr-1*. Motility was quantified by measuring the diameter of the motility halo after incubation at 37°C for 30 h. (**D**) Biofilm formation of WT, TC, TCΔ*mcr-1*, and TCΔ*mcr-1*+p*mcr-1*. Biofilm biomass was quantified by crystal violet staining after incubation at 37°C for 72 h. Representative images of crystal violet-stained biofilms are shown above the graph. Data in panels **B–D** represent the mean ± standard deviation from three independent experiments. Statistical significance in panels **C** and **D** was determined using one-way ANOVA followed by Tukey’s multiple-comparisons test. *, *P* < 0.05; **, *P* < 0.01.

A plasmid stability test was conducted to determine whether the *mcr-1* plasmid transferred by conjugation was stably maintained in the recipient strain, *E. coli* ATCC 43894. FORC82, the original *mcr-1*-harboring strain, was used as the control. No plasmid loss was detected in either strain during 10 consecutive subcultures, indicating that the *mcr-1* plasmid was stably maintained in ATCC 43894 (Fig. 4A). In addition, no significant difference in growth was observed between the wild-type and the transconjugant (Fig. 4B). The colistin minimum inhibitory concentration (MIC) increased from 1 μg/mL in the wild-type strain to 2 μg/mL in the transconjugant, decreased to 1 μg/mL in the Δ*mcr-1* mutant, and was restored to 2 μg/mL in the *mcr-1* complemented strain (Table S3).

To investigate whether *mcr-1* was responsible for the altered phenotypes, we constructed ∆*mcr-1* mutant and *mcr-1* complemented strains. Swimming motility was restored in the ∆*mcr-1* mutant strain to the wild-type level, and motility was reduced in the *mcr-1* complemented strain compared with the ∆*mcr-1* mutant strain (Fig. 4C). Notably, biofilm formation in the ∆*mcr-1* mutant strain was restored to wild-type levels, whereas biofilm formation in the *mcr-1* complemented strain was reduced to the level observed in the transconjugant (Fig. 4D). These results indicate that *mcr-1* is responsible for the reduced swimming motility and biofilm formation observed in ATCC 43894.

### Acquisition of *mcr-1* alters cell surface properties and adhesion

**Fig 5:**
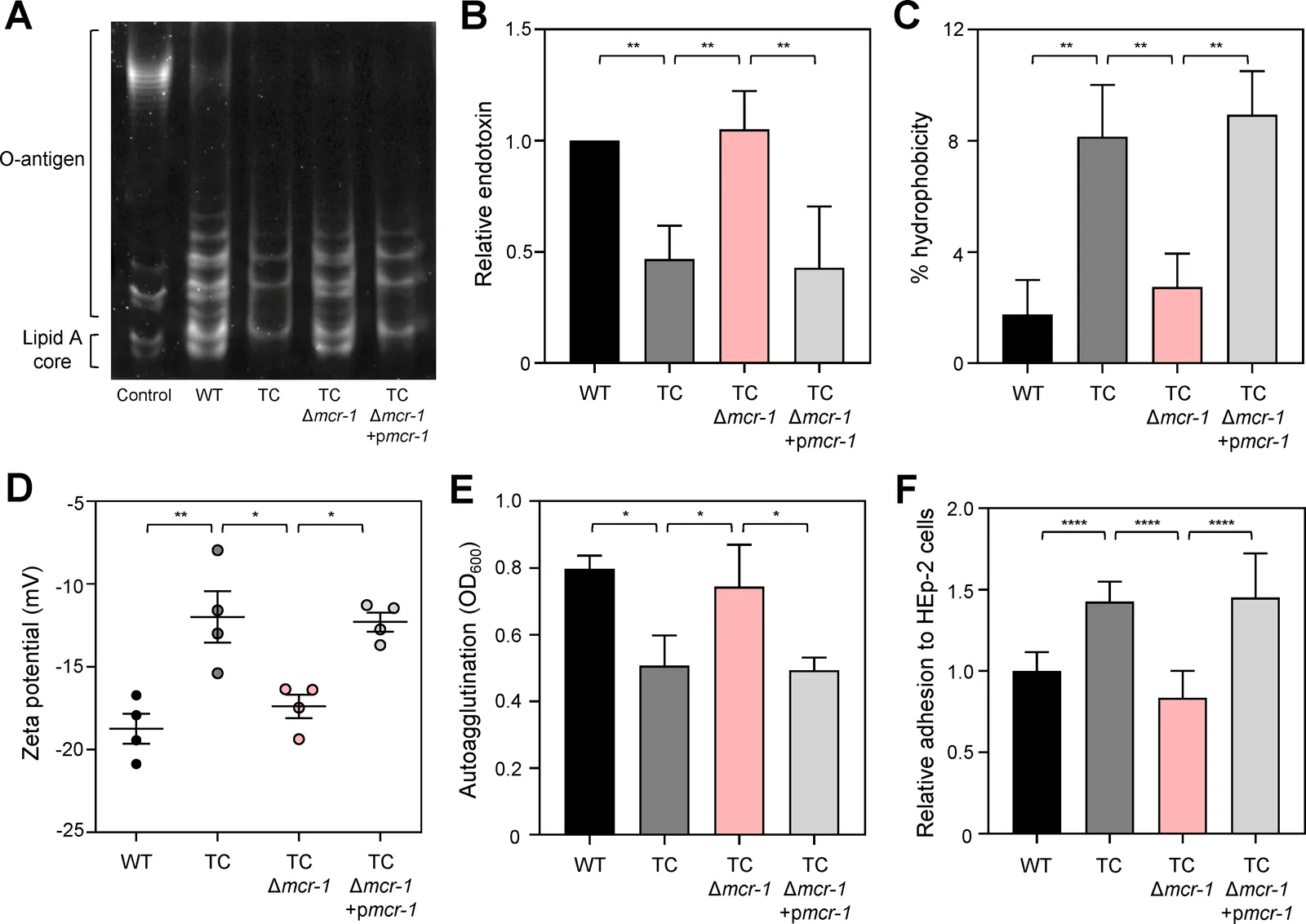
*mcr-1* alters cell surface properties and adhesion in *E. coli* O157:H7 ATCC 43894. (**A**) LPS profiles of the wild-type strain (WT), transconjugant (TC), Δ*mcr-1* mutant (TCΔ*mcr-1*), and *mcr-1* complemented strain (TCΔ*mcr-1*+p*mcr-1*). LPS extracts were analyzed by DOC-PAGE. (**B**) Relative endotoxin levels of WT, TC, TCΔ*mcr-1*, and TCΔ*mcr-1*+p*mcr-1*. Endotoxin levels were measured using the Limulus amebocyte lysate assay and are presented relative to those of the WT strain. (**C**) Cell surface hydrophobicity of WT, TC, TCΔ*mcr-1*, and TCΔ*mcr-1*+p*mcr-1*. Hydrophobicity was determined by measuring bacterial adhesion to n-hexadecane and is expressed as percentage hydrophobicity. (**D**) Zeta potential of WT, TC, TCΔ*mcr-1*, and TCΔ*mcr-1*+p*mcr-1*. Each symbol represents an independent biological replicate, and horizontal lines indicate the mean. (**E**) Autoagglutination of WT, TC, TCΔ*mcr-1*, and TCΔ*mcr-1*+p*mcr-1*. Autoagglutination was quantified by measuring the OD_600_ of the upper suspension after 1 h of incubation. (**F**) Adhesion of WT, TC, TCΔ*mcr-1*, and TCΔ*mcr-1*+p*mcr-1* to HEp-2 cells. HEp-2 monolayers were infected with bacteria at an MOI of 10 and incubated for 3 h at 37°C. Adhesion is presented relative to that of the WT. Data in panels **B–F** represent the mean ± standard deviation from three independent experiments. Statistical significance in panels **B–F** was determined using one-way ANOVA followed by Tukey’s multiple comparisons test. *, *P* < 0.05; **, *P* < 0.01; ****, *P* < 0.0001.

Because *mcr-1* is associated with lipid A modification, we investigated whether acquisition of the *mcr-1*-harboring plasmid altered the cell surface properties of ATCC 43894. LPS was extracted and analyzed by deoxycholate-polyacrylamide gel electrophoresis (DOC-PAGE). The transconjugant exhibited an altered LPS profile, characterized by reduced bands in the O-antigen region and the core-lipid A region compared with the wild-type strain (Fig. 5A). These alterations were abolished in the Δ*mcr-1* mutant strain and restored in the *mcr-1* complemented strain (Fig. 5A). Consistent with the altered LPS profile, the relative endotoxin level was significantly reduced in the transconjugant, recovered in the Δ*mcr-1* mutant, and decreased again in the *mcr-1* complemented strain (Fig. 5B).

These changes in the LPS profile were accompanied by broader alterations in cell surface-associated phenotypes. Cell surface hydrophobicity was significantly increased in the transconjugant compared with the wild-type strain, whereas the Δ*mcr-1* mutant showed a restored phenotype and the *mcr-1* complemented strain again exhibited increased hydrophobicity (Fig. 5C). The transconjugant showed a less negative zeta potential than the wild-type strain, indicating that *mcr-1* reduced the negative surface charge of ATCC 43894. This change was reversed in the Δ*mcr-1* mutant strain and restored by complementation (Fig. 5D). Autoagglutination was also reduced by *mcr-1*, as the transconjugant showed lower autoagglutination than the wild-type strain, whereas this phenotype was reversed in the Δ*mcr-1* mutant strain and restored following complementation (Fig. 5E). In addition, adhesion to HEp-2 cells was reduced in the Δ*mcr-1* mutant strain and restored in the *mcr-1* complemented strain to a level comparable to that of the transconjugant (Fig. 5F). Together, these results indicate that *mcr-1*-mediated remodeling of the LPS profile is accompanied by broad changes in cell surface properties and enhanced epithelial cell adhesion.

## DISCUSSION

The emergence of plasmid-mediated colistin resistance in STEC raises concerns beyond the loss of a last-resort antibiotic because *mcr-1* acquisition may bring both broader resistance co-selection and physiological changes to an already challenging foodborne pathogen. Although *mcr*-harboring STEC has been increasingly reported from food-producing animals and retail meat (14, 16), the phenotypic consequences of *mcr-1* acquisition in a STEC background remain largely unexamined. We previously showed in enterotoxigenic *E. coli* that *mcr-1*-harboring plasmids upregulate surface polysaccharide biosynthesis and drive a transition from a rough to a smooth LPS phenotype, accompanied by enhanced virulence (18). Whether and how *mcr-1* acquisition affects the surface architecture of STEC, which already produces a smooth, O-antigen-bearing LPS, has not been examined. Here, we extend that work to STEC and show that *mcr-1* acquisition drives extensive co-resistance, LPS remodeling, and broad changes in cell surface properties and host-cell interaction.

Among the 144 *mcr*-harboring STEC isolates, *mcr-1* was the predominant variant (63.9%), with *mcr-1.1* accounting for 86.9% of *mcr-1*-positive isolates. This mirrors the global dominance of *mcr-1.1* in *E. coli* (2, 6). The minor variants *mcr-1.32* and *mcr-1.5* showed source-biased distribution, with *mcr-1.5* exclusive to pig isolates and *mcr-1.32* enriched in human isolates, suggesting host-associated selection. The Clermont phylogroup distribution also differed across pathotype categories. Phylogroup A is generally associated with commensal and enterotoxigenic *E. coli* (19), and its strong representation among ETEC/STEC isolates may reflect ETEC-like backbones acquiring *stx* via phage transfer. The recovery of *mcr-1*-harboring STEC across multiple phylogenetic backgrounds and source categories indicates that *mcr-1* has disseminated broadly along the food chain.

A noteworthy feature of our data set is the predominance of pig-derived isolates among *mcr-1*-positive STEC (66 of 99 isolates, 66.7%), in contrast to the conventional view of cattle and other ruminants as the principal STEC reservoirs (20). Two factors likely contribute to this pattern. First, swine have been increasingly recognized as substantial STEC carriers, with high rates of *stx*-positive carriage reported among finishing-stage pigs across multiple regions (21, 22). Second, the global emergence and spread of *mcr-1* have been most strongly driven by colistin use in food-producing animals, particularly in swine production, where colistin was widely used as a growth promoter and metaphylactic agent before its restriction in many countries (6, 23). The convergence of pigs as both STEC carriers and the principal selective environment for *mcr-1* provides a parsimonious explanation for the pig predominance among *mcr-1*-positive STEC observed here. Additionally, we acknowledge that database-level sampling bias cannot be excluded, since the isolates available through NCBI reflect the activities of regional surveillance programs and outbreak investigations rather than a random sample of the global STEC population.

A particularly concerning feature was the high prevalence of co-resistance to other antimicrobial classes, with 88.9% of isolates classified as multidrug-resistant. This co-resistance pattern is clinically important because antibiotic therapy of STEC infection is already constrained by the fact that antimicrobial agents that activate the bacterial SOS response induce the lambdoid prophages carrying *stx* genes and increase Stx production, which has been linked to a higher risk of HUS (11, 12, 24). In this restricted therapeutic landscape, fosfomycin has attracted attention as a comparatively favorable option because early fosfomycin treatment may reduce progression to HUS (13). Against this background, our finding that genomically predicted fosfomycin resistance determinants were present in 84.8% of *mcr-1*-positive STEC isolates is striking.

Chromosomal substitutions in GlpT and UhpT dominated, with the GlpT_E448K substitution alone or in combination with UhpT_E350Q accounting for 78 of the 99 isolates (78.8%). GlpT and UhpT are the glycerol-3-phosphate and hexose-6-phosphate transporters of *E. coli*, respectively, and substitutions in these transporters are a recognized chromosomal mechanism of reduced fosfomycin susceptibility (25, 26), including in STEC, where concurrent alterations in *glpT* and *uhpT* have been reported (27). In addition, primarily plasmid-mediated fosfomycin-modifying enzyme genes (*fosA3*, *fosA7.5*, and *fosA*) were detected in three isolates (3.0%). Although infrequent, the co-occurrence of *fosA*-family genes with *mcr-1* in the same STEC genome indicates that mobile fosfomycin resistance can also enter the *mcr-1*-positive STEC population. This overall prevalence of genomically predicted fosfomycin resistance determinants is markedly higher than previously reported rates of phenotypic fosfomycin resistance in pig-associated *E. coli* (28). The dominant determinants in our data set, the chromosomal *glpT* and *uhpT* substitutions, are not co-transferred with the *mcr-1* plasmid, so the high co-prevalence reflects host strain-level co-occurrence rather than physical genetic linkage, and phenotypic confirmation by MIC determination will be required to translate the genomic signal into a clinical resistance claim. Even with these caveats, *mcr-1*-positive STEC isolates simultaneously carry determinants associated with reduced susceptibility to two of the most relevant remaining therapeutic options for severe STEC infection.

Although acquisition of *mcr-1* increased the colistin MIC from 1 to 2 μg/mL, this value is at the EUCAST epidemiological cutoff for *E. coli* (29). The level of *mcr-1*-mediated colistin resistance can vary, and *mcr-1*-positive *E. coli* with a colistin MIC of 2 μg/mL, including heteroresistant populations, have been reported (30, 31). Thus, the observed MIC increase indicates a functional effect of the *mcr-1* resistance determinant on colistin susceptibility rather than acquisition of a phenotypically resistant state. Phenotypically, *mcr-1* acquisition in *E. coli* O157:H7 ATCC 43894 produced a coordinated reshaping of cell surface properties. The transconjugant exhibited an altered LPS profile, a less negative zeta potential, increased hydrophobicity, reduced autoagglutination, and reduced endotoxin activity, all of which were reversed in the Δ*mcr-1* mutant and restored upon complementation. These changes are consistent with the established activity of MCR-1, which transfers phosphoethanolamine to lipid A and reduces the net negative charge of the outer membrane (3). Lipid A remodeling also attenuates TLR4-mediated recognition of LPS (32), which provides a parsimonious explanation for the reduced *G. mellonella* mortality observed after infection with the transconjugant, given that this insect model is largely driven by innate-immune recognition of bacterial surface molecules. We therefore caution against interpreting the *G. mellonella* result as a generalized loss of virulence. The same surface remodeling that limits innate-immune detection also enhanced adhesion of the transconjugant to HEp-2 epithelial cells. In a foodborne pathogen whose disease begins with intestinal colonization, increased epithelial adhesion is the more directly relevant phenotype, and the apparent dichotomy between insect-model survival and epithelial-cell interaction is best understood as two facets of a single underlying surface-remodeling event.

The mechanism by which *mcr-1* affects motility and biofilm formation in *E. coli* O157:H7 remains to be defined. An interesting precedent exists in *Campylobacter jejuni*, where the phosphoethanolamine transferase EptC, a member of the same superfamily as MCR-1, modifies both lipid A and the flagellar rod protein FlgG; however, the FlgG modification, rather than the lipid A modification, was specifically shown to be required for full motility (33). Whether MCR-1 has analogous secondary substrates in *E. coli*, or whether the reduced motility in our transconjugant arises indirectly from LPS-mediated changes in outer-membrane integrity, will require dedicated mechanistic study. Reduced biofilm formation may be associated with altered LPS structure and outer-membrane charge, which may affect cell-cell aggregation in *E. coli*. Reduced motility and biofilm in the transconjugant do not necessarily imply reduced fitness in the food chain. STEC commonly transitions between motile colonization phases and sessile reservoir niches, and an *mcr-1*-driven shift toward stronger epithelial adhesion combined with reduced motility and biofilm may represent a reallocation of host-interaction strategy rather than an attenuation. Notably, the LPS phenotype observed in this study is opposite in direction to what we previously reported for the same plasmid in enterotoxigenic *E. coli*, in which *mcr-1* acquisition drove a rough-to-smooth LPS transition through upregulation of surface polysaccharide biosynthesis and was accompanied by enhanced virulence in a murine model (18). In *E. coli* O157:H7 ATCC 43894, which already produces a smooth, O-antigen-bearing LPS, *mcr-1* acquisition was instead associated with reduced LPS banding in both the O-antigen and core-lipid A regions. The contrasting outcomes indicate that the phenotypic consequence of *mcr-1* acquisition is strongly shaped by the recipient strain’s pre-existing LPS architecture, and that observations from one *E. coli* background should not be uncritically generalized to STEC.

This study has several limitations. The phenotypic experiments were performed in a single STEC reference strain, and confirmation in additional STEC backgrounds will be needed. The complementation construct was based on a high-copy pUC19 backbone, and dosage effects on the more sensitive surface phenotypes should be addressed using single-copy chromosomal complementation. Stx production and prophage induction were not assessed in this study, and given the SOS-coupled regulation of Stx-encoding prophages (34), determining whether *mcr-1* acquisition modulates Stx output is a clear next step. Finally, the genomic association between *mcr-1* and fosfomycin resistance determinants will need to be confirmed by direct MIC measurement.

## MATERIALS AND METHODS

### Bacterial strains and growth conditions

*E. coli* O157:H7 strain ATCC 43894 was used as the wild-type strain in this study. The *mcr-1* plasmid (pFORC82_3) used in this study was originally identified in *E. coli* FORC82, isolated from raw chicken (35). Analysis of pFORC82_3 using ResFinder identified *mcr-1* as the only antimicrobial resistance gene, while no known virulence factors were detected using the VFDB core data set. All *E. coli* strains were cultured in Luria–Bertani (LB) medium (Difco, USA) at 37°C. When required, antibiotics and chemicals were added to the medium at final concentrations of 2 µg/mL colistin, 50 µg/mL ampicillin, and 0.25 mM isopropyl-β-D-thiogalactopyranoside.

### Strain construction

The primers used in this study are listed in Table S1. To construct the transconjugant, the *mcr-1* plasmid from FORC82 was transferred into the wild-type strain by conjugation. The Δ*mcr-1* mutant strains were generated by lambda Red recombination (36). To construct the *mcr-1* complemented strain, the coding sequence of *mcr-1* was PCR-amplified from pFORC82_3 and cloned into pUC19. The recombinant plasmid was introduced into the Δ*mcr-1* mutant strain. All constructed strains were verified by Sanger sequencing of the targeted gene (Bionics, Seoul, South Korea).

### Identification and genomic characterization of *mcr*-harboring STEC isolates

*mcr*-harboring STEC isolates were identified using the NCBI Pathogen Detection Isolates Browser (https://www.ncbi.nlm.nih.gov/pathogens/). The database was searched using the query (*stx1* OR *stx2*) AND *mcr* to retrieve isolates carrying *stx* genes together with any member of the *mcr* gene family. From the initial search results, isolates with publicly available raw sequencing reads in FASTQ format were selected for further analysis. After removal of duplicate or incomplete entries, a total of 144 *mcr*-harboring STEC-associated isolates were included in this study. The selected isolates were analyzed using EnteroBase within the *Escherichia*/*Shigella* database. Raw sequencing reads were uploaded to EnteroBase and processed through the automated assembly and genotyping pipeline. Genomic features, including the presence of *mcr* genes, *mcr-1* variants, *stx* genes, enterotoxin genes, eae, antimicrobial resistance genes, and Clermont phylogroups, were retrieved from the EnteroBase output. Isolate metadata, including host or source information, were obtained from the corresponding NCBI records.

For pathovar classification, isolates carrying *stx1* and/or *stx2* were classified as STEC-associated isolates. Isolates carrying both *stx* genes and enterotoxin genes, including *st* and/or *lt*, were classified as ETEC/STEC. Isolates carrying *stx* genes together with *eae* were classified as EHEC. When isolates carried overlapping virulence gene profiles, classification was assigned based on the combination of major pathotype-associated markers detected in the genome. Antimicrobial resistance gene profiles were summarized by antibiotic class, and the proportion of isolates carrying resistance genes in each class was calculated among the *mcr-1*-harboring isolate subset. Chromosomal mutations associated with fosfomycin resistance, including substitutions in the glycerol-3-phosphate (GlpT) and hexose-6-phosphate (UhpT) transporters that mediate fosfomycin uptake into *E. coli* (25), were retrieved from the EnteroBase output.

### Cell adhesion assay

The adhesion assay using HEp-2 cells was performed as described previously (37). Briefly, HEp-2 cells were maintained in Eagle’s minimal essential medium (EMEM) supplemented with 10% fetal bovine serum (FBS) at 37°C under 5% CO_2_. A monolayer of 4 × 10^5^ HEp-2 cells was prepared in a 24-well tissue culture plate. Then, 150 µL of overnight culture was inoculated into 3 mL of LB broth and incubated at 37°C until the OD_600_ reached 1.0. The bacterial suspension was diluted in EMEM supplemented with 10% FBS and added to the cell monolayer at an MOI of 10. After 3 h of incubation at 37°C, the cells were washed three times with prewarmed PBS to remove nonadherent bacteria, lysed with 1% Triton X-100 for 10–15 min, and serially diluted in PBS. The suspensions were plated on LB agar plates to enumerate colony-forming units (CFUs).

### Swimming motility assay

The swimming motility assay was performed as previously described (18). Overnight cultures were diluted in LB broth to an OD_600_ of 0.6, and 2 μL of each diluted culture was inoculated into swimming motility agar plates containing 0.3% agar. After incubation for 30 h at 37°C, the diameters of the motility halos were measured.

### Biofilm formation assay

The biofilm formation assay was performed as previously described (18, 38). Overnight cultures were diluted in LB broth to an OD_600_ of 0.05, inoculated into a 96-well plate, and incubated for 72 h at 37°C. After incubation, the wells were washed three times with PBS, stained with 0.1% crystal violet for 20 min, and destained with 33% acetic acid. The absorbance was then measured at 570 nm using a SpectraMax i3 multimode microplate reader (Molecular Devices, CA, USA).

### *Galleria mellonella* infection model

The *Galleria mellonella* infection assay was performed as previously described (18). Briefly, overnight cultures were diluted 1:100 in 10 mL of LB broth and incubated at 37°C until the OD_600_ reached 1.0. Bacterial cells were collected by centrifugation at 16,000 *× g* for 1 min at 4°C, washed, and resuspended in PBS to 1 × 10^6^ CFU/mL. Each larva weighing 200–250 mg was injected with 10 μL of the bacterial suspension into a proleg using a 50 μL Hamilton syringe. Following injection, the larvae were incubated at 37°C and monitored for survival over 72 h. Larvae injected with PBS served as the negative control, and no mortality was observed in this group.

### Plasmid stability test

The plasmid stability test was performed as described previously (18). Briefly, transconjugants were subcultured into fresh LB broth without colistin for 10 consecutive days. After each 24 h incubation, cultures were serially diluted and plated on LB agar, followed by overnight incubation at 37°C. To determine plasmid retention, 96 colonies from each culture were transferred to 96-well plates containing LB broth supplemented with 4 µg/mL colistin, and the proportion of plasmid-containing cells was calculated based on the number of wells showing visible growth. FORC82, the original *mcr-1*-harboring strain, was used as the control.

### Growth curve

Overnight cultures were diluted 1:100 into fresh LB broth and monitored every 30 min for 12 h at 37°C. Growth rates were determined by measuring the OD_600_ using a SpectraMax i3 multimode microplate reader (Molecular Devices, CA, USA).

### Antimicrobial susceptibility testing

The MIC of colistin was determined by the gradient diffusion method using E-test strips (Liofilchem, Italy). Bacterial suspensions were adjusted to a 0.5 McFarland standard and inoculated onto Mueller-Hinton agar plates. Colistin E-test strip (0.016–256 μg/mL) was placed on the inoculated plates, which were then incubated at 37°C for 16–20 h. MIC values were determined at the point where the inhibition ellipse intersected the strip, according to the manufacturer’s instructions.

### LPS extraction, analysis, and endotoxin quantification

LPS was extracted using a hot phenol-water micro-extraction method as described previously (18, 39). The extracted LPS were analyzed by DOC-PAGE, and the gels were stained using the Pro-Q Emerald 300 Lipopolysaccharide Gel Stain Kit (cat. no. P20495, Molecular Probes, OR, USA) according to the manufacturer’s instructions. Endotoxin levels of the extracted LPS were determined using the Pierce LAL Chromogenic Endotoxin Quantitation Kit (Thermo Scientific, cat. no. A39552) according to the manufacturer’s instructions.

### Cell surface hydrophobicity

The cell surface hydrophobicity was measured as previously described (18), with minor modifications. Briefly, overnight cultures were harvested by centrifugation at 16,000 *× g* for 1 min at 4°C and washed with PBS. The bacterial suspension was diluted to an OD_600_ of 1 with PBS, and the OD_600_ was measured. Then 200 µL of n-hexadecane was added to the suspension, and the mixture was vortexed for 2 min. The mixture was allowed to rest for 1 h at room temperature to achieve phase separation, and the OD_600_ of the lower aqueous phase was measured. The cell hydrophobicity was calculated.

### Zeta potential measurement

The zeta potential was measured as previously described (18). Briefly, overnight cultures were diluted 1:100 in 10 mL of LB broth and incubated at 37°C until the OD_600_ reached 1.0. Bacterial cells were harvested by centrifugation at 5,000 *× g* for 5 min at 4°C, washed twice with ddH_2_O, and resuspended in 1 mL of ddH_2_O. The zeta potential was then measured using a Zetasizer Nano ZS90 analyzer (Malvern Panalytical, Malvern, UK).

### Autoagglutination assay

Overnight cultures were diluted 1:100 in 10 mL of LB broth and incubated at 37°C until the OD_600_ reached 1.0. The cultures were transferred to tubes and allowed to stand for 1 h at room temperature. The OD_600_ of the upper suspension was then measured.

### Statistical analysis

Statistical analyses were conducted using GraphPad Prism (version 8.0.1, USA). Data are presented as the mean ± standard deviation from at least three independent biological replicates. Statistical significance was determined using an unpaired two-tailed *t*-test or one-way ANOVA followed by Tukey’s multiple comparisons test. Survival curves were analyzed using the Kaplan–Meier method and compared using the log-rank (Mantel–Cox) test. *, *P*  < 0.05; **, *P*  < 0.01; ***, *P* <  0.001; and ****, *P* <  0.0001.

## Data Availability

All data supporting the findings of this study are available within the article and its supplemental material. The publicly available sequencing data analyzed in this study were retrieved from the NCBI Pathogen Detection database (https://www.ncbi.nlm.nih.gov/pathogens/), and the corresponding BioSample accession numbers are listed in Table S2.

## SUPPLEMENTAL MATERIAL

Supplemental tables spectrum.02034-26-s0001.docx Tables S1 and S3.

Table S2 spectrum.02034-26-s0002.xlsx Genomic characteristics, source metadata, and antimicrobial resistance profiles of 144 mcr-harboring STEC isolates.

ASM does not own the copyrights to Supplemental Material that may be linked to, or accessed through, an article. The authors have granted ASM a non-exclusive, world-wide license to publish the Supplemental Material files. Please contact the corresponding author directly for reuse.
