# Supplementary material for: Multidrug resistance and lipopolysaccharide remodeling associated with mcr-1 acquisition in Shiga toxin-producing Escherichia coli
Source: Microbiol Spectr. 2026 Sep 30;14(11):e02034-26. doi: 10.1128/spectrum.02034-26 (PMC13729178; doi:10.1128/spectrum.02034-26)
Supplement: Supplementary file 1 [file spectrum.02034-26-s0001.docx]

**Table S1. Primers used in this study**

| **Primer** | **Sequence (5′-3′)** | **Reference** |
| --- | --- | --- |
| mcr-1-P1-Kan-F | ATGGAGTGTGCGGTGGGTTTGGAAAAAATACAGGGAGAAATGTAGGCTGGAGCTGCTTCG | This study |
| mcr-1-P4-Kan-R | GCCGCAATTATCCCACCGTTTATTTTTTGAGTAGTTTCTCCTGTCAAACATGAGAATTAATTCC |  |
| mcr-1-con-F | CATCTCAGCAAGTAGGCGTT | (Ahn et al., 2025) |
| mcr-1-con-R | GAATTGCCGCAATTATCCCAC |  |
| Kan-con-F | GCAAGGTGAGATGACAGGAG | This study |
| Kan-con-R | CGACCACCA AGCGAAACATC |  |
| SalI-mcr-1-F | TTGTCGACATGATGCAGCATACTTCTGTGT |  |
| EcoRI-mcr-1-R | AAGAATTCGGGAGAAATCAGCGGATGAA |  |
|  |  |  |

**Table S3.** MIC of colistin

| **Strains^a^** | **MIC (mg/L)** |
| --- | --- |
| WT+pUC19 | 1 |
| TC+pUC19 | 2 |
| TCΔ*mcr-1*+pUC19 | 1 |
| TCΔ*mcr-1*+pUC19::*mcr-1* | 2 |

^a^WT, wild type (*E. coli* O157:H7 ATCC 43894); TC, transconjugant.

**References**

Ahn, E., Kim, J., Jiang, J., Kim, J., Muszyński, A., Kasperkiewicz, K., Shin, H., Cao, Y., Soh, Y., Park, Y., 2025. Colistin resistance plasmids dually enhance bacterial virulence and antibiotic resistance via surface polysaccharide biosynthesis. Nat. Commun. 16, 9966. <https://doi.org/10.1038/s41467-025-65412-9>
